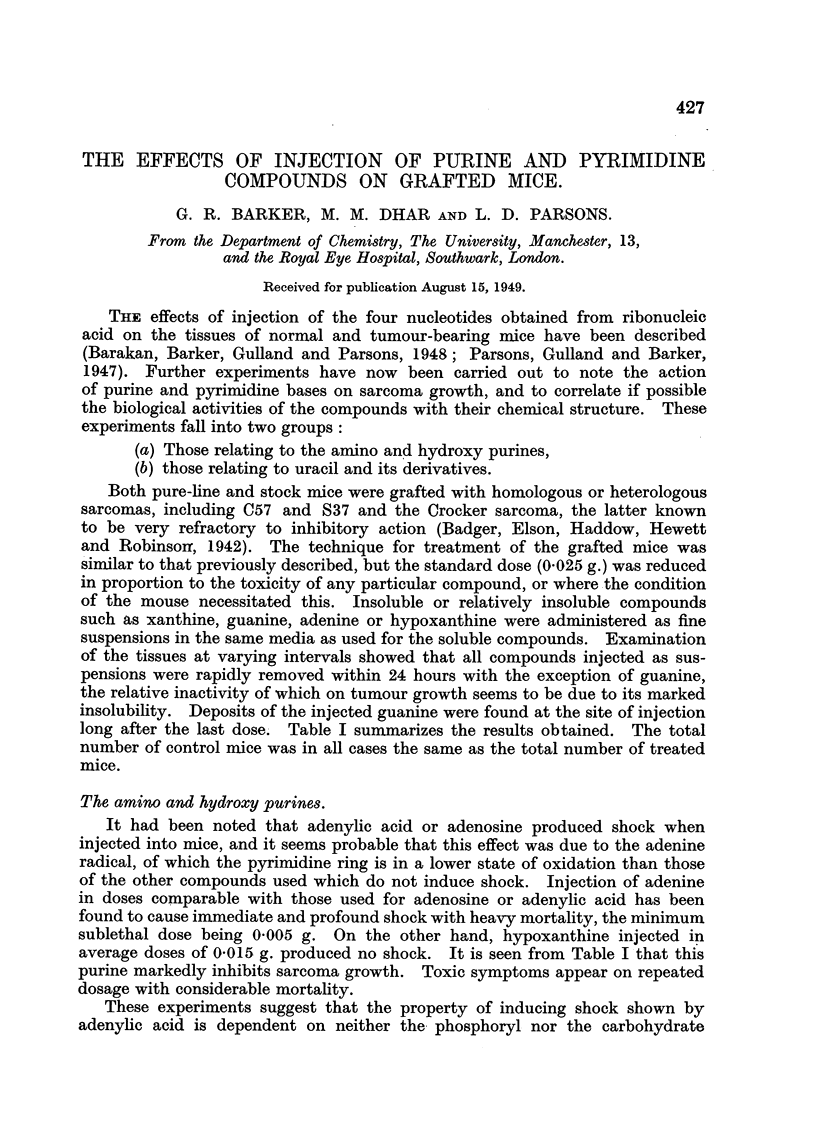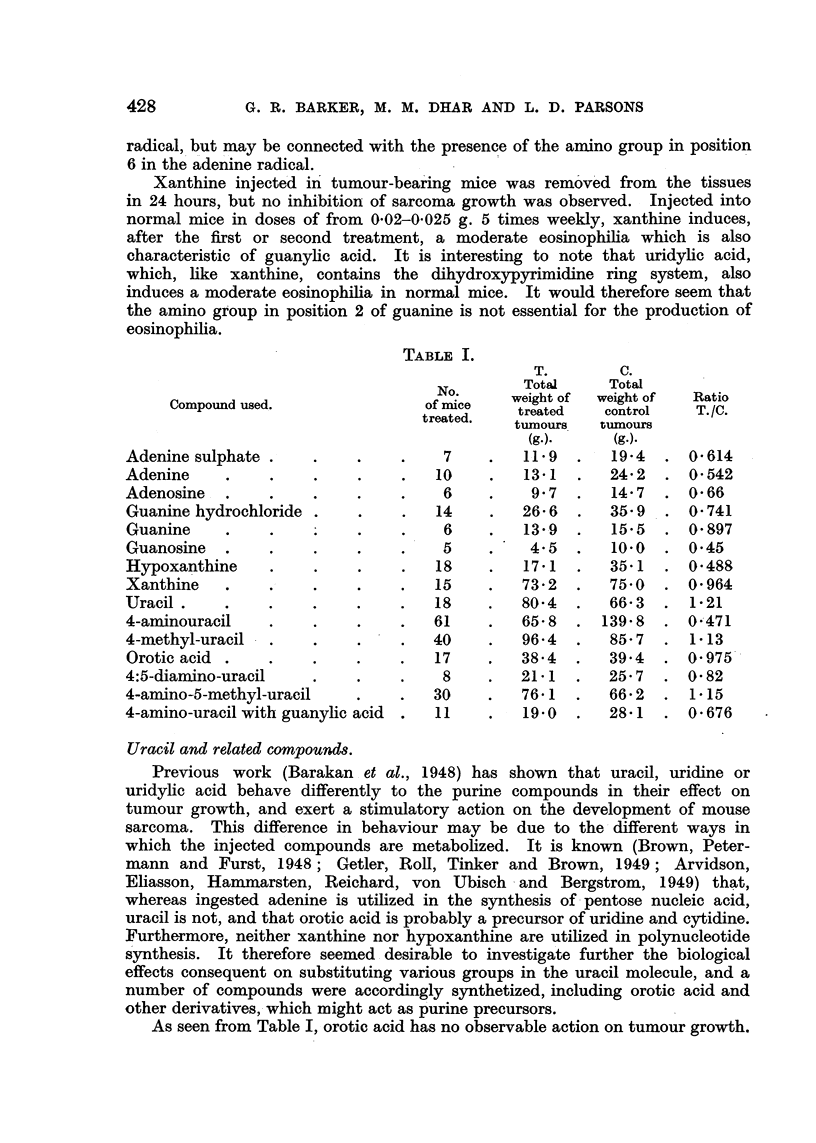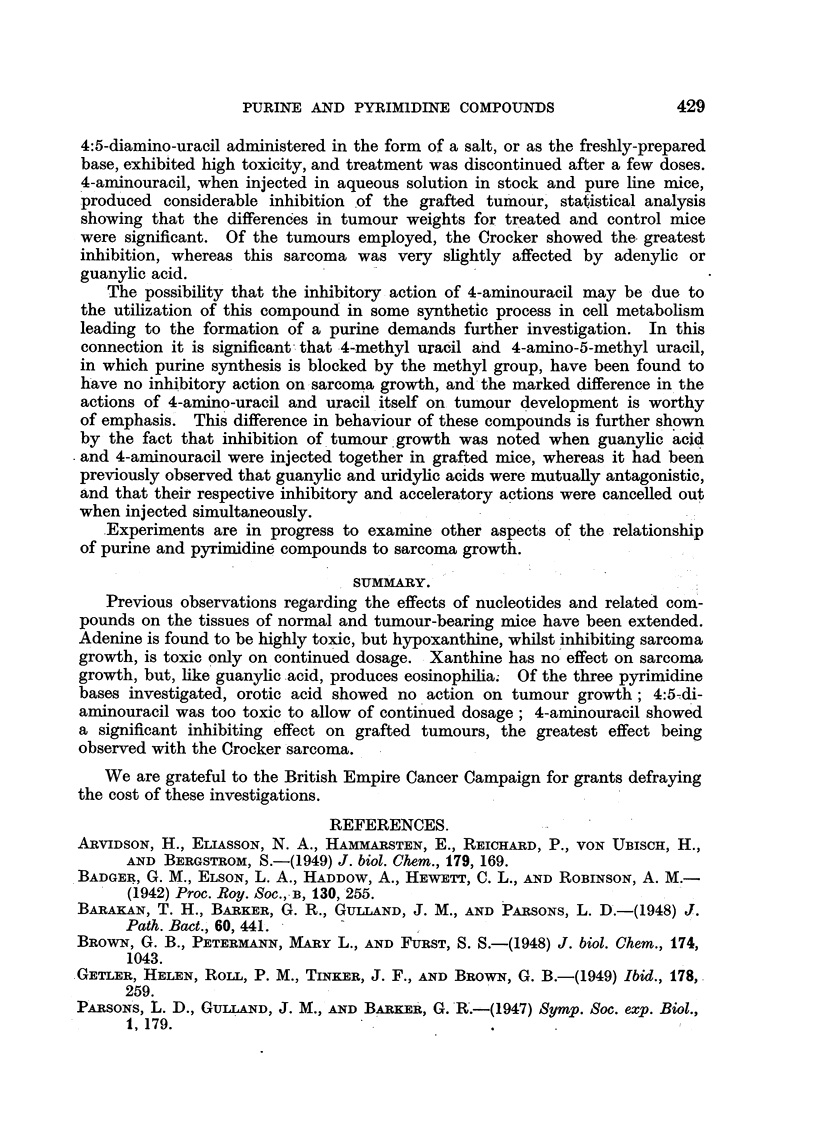# The Effects of Injection of Purine and Pyrimidine Compounds on Grafted Mice

**DOI:** 10.1038/bjc.1949.47

**Published:** 1949-09

**Authors:** G. R. Barker, M. M. Dhar, L. D. Parsons


### THE EFFECTS OF INJECTION OF PURINE AND PYRIMIDINE COMPOUNDS ON GRAFTED MICE.

G. R. BARKER, M. M. DHAR AND L. D. PARSONS.

*From the Department of Chemistry, The University, Manchester, 13, and the Royal Eye Hospital, Southwark, London.*

Received for publication August 15, 1949.

THE effects of injection of the four nucleotides obtained from ribonucleic acid on the tissues of normal and tumour-bearing mice have been described (Barakan, Barker, Gulland and Parsons, 1948; Parsons, Gulland and Barker, 1947). Further experiments have now been carried out to note the action of purine and pyrimidine bases on sarcoma growth, and to correlate if possible the biological activities of the compounds with their chemical structure. These experiments fall into two groups :

(*a*) Those relating to the amino and hydroxy purines,
(*b*) those relating to uracil and its derivatives.

Both pure-line and stock mice were grafted with homologous or heterologous sarcomas, including C57 and S37 and the Crocker sarcoma, the latter known to be very refractory to inhibitory action (Badger, Elson, Haddow, Hewett and Robinson, 1942). The technique for treatment of the grafted mice was similar to that previously described, but the standard dose (0·025 g.) was reduced in proportion to the toxicity of any particular compound, or where the condition of the mouse necessitated this. Insoluble or relatively insoluble compounds such as xanthine, guanine, adenine or hypoxanthine were administered as fine suspensions in the same media as used for the soluble compounds. Examination of the tissues at varying intervals showed that all compounds injected as suspensions were rapidly removed within 24 hours with the exception of guanine, the relative inactivity of which on tumour growth seems to be due to its marked insolubility. Deposits of the injected guanine were found at the site of injection long after the last dose. Table I summarizes the results obtained. The total number of control mice was in all cases the same as the total number of treated mice.

*The amino and hydroxy purines.*

It had been noted that adenylic acid or adenosine produced shock when injected into mice, and it seems probable that this effect was due to the adenine radical, of which the pyrimidine ring is in a lower state of oxidation than those of the other compounds used which do not induce shock. Injection of adenine in doses comparable with those used for adenosine or adenylic acid has been found to cause immediate and profound shock with heavy mortality, the minimum sublethal dose being 0·005 g. On the other hand, hypoxanthine injected in average doses of 0·015 g. produced no shock. It is seen from Table I that this purine markedly inhibits sarcoma growth. Toxic symptoms appear on repeated dosage with considerable mortality.

These experiments suggest that the property of inducing shock shown by adenylic acid is dependent on neither the phosphoryl nor the carbohydrate radical, but may be connected with the presence of the amino group in position 6 in the adenine radical.

Xanthine injected in tumour-bearing mice was removed from the tissues in 24 hours, but no inhibition of sarcoma growth was observed. Injected into normal mice in doses of from 0·02–0·025 g. 5 times weekly, xanthine induces, after the first or second treatment, a moderate eosinophilia which is also characteristic of guanylic acid. It is interesting to note that uridylic acid, which, like xanthine, contains the dihydroxypyrimidine ring system, also induces a moderate eosinophilia in normal mice. It would therefore seem that the amino group in position 2 of guanine is not essential for the production of eosinophilia.

TABLE I.

| Compound used. | No. of mice treated. | T. Total weight of treated tumours (g.). | C. Total weight of control tumours (g.). | Ratio T./C. |
|---|---|---|---|---|
| Adenine sulphate | 7 | 11·9 | 19·4 | 0·614 |
| Adenine | 10 | 13·1 | 24·2 | 0·542 |
| Adenosine | 6 | 9·7 | 14·7 | 0·66 |
| Guanine hydrochloride | 14 | 26·6 | 35·9 | 0·741 |
| Guanine | 6 | 13·9 | 15·5 | 0·897 |
| Guanosine | 5 | 4·5 | 10·0 | 0·45 |
| Hypoxanthine | 18 | 17·1 | 35·1 | 0·488 |
| Xanthine | 15 | 73·2 | 75·0 | 0·964 |
| Uracil | 18 | 80·4 | 66·3 | 1·21 |
| 4-aminouracil | 61 | 65·8 | 139·8 | 0·471 |
| 4-methyl-uracil | 40 | 96·4 | 85·7 | 1·13 |
| Orotic acid | 17 | 38·4 | 39·4 | 0·975 |
| 4:5-diamino-uracil | 8 | 21·1 | 25·7 | 0·82 |
| 4-amino-5-methyl-uracil | 30 | 76·1 | 66·2 | 1·15 |
| 4-amino-uracil with guanylic acid | 11 | 19·0 | 28·1 | 0·676 |

*Uracil and related compounds.*

Previous work (Barakan *et al.*, 1948) has shown that uracil, uridine or uridylic acid behave differently to the purine compounds in their effect on tumour growth, and exert a stimulatory action on the development of mouse sarcoma. This difference in behaviour may be due to the different ways in which the injected compounds are metabolized. It is known (Brown, Petermann and Furst, 1948; Getler, Roll, Tinker and Brown, 1949; Arvidson, Eliasson, Hammarsten, Reichard, von Ubisch and Bergstrom, 1949) that, whereas ingested adenine is utilized in the synthesis of pentose nucleic acid, uracil is not, and that orotic acid is probably a precursor of uridine and cytidine. Furthermore, neither xanthine nor hypoxanthine are utilized in polynucleotide synthesis. It therefore seemed desirable to investigate further the biological effects consequent on substituting various groups in the uracil molecule, and a number of compounds were accordingly synthetized, including orotic acid and other derivatives, which might act as purine precursors.

As seen from Table I, orotic acid has no observable action on tumour growth.

4:5-diamino-uracil administered in the form of a salt, or as the freshly-prepared base, exhibited high toxicity, and treatment was discontinued after a few doses. 4-aminouracil, when injected in aqueous solution in stock and pure line mice, produced considerable inhibition of the grafted tumour, statistical analysis showing that the differences in tumour weights for treated and control mice were significant. Of the tumours employed, the Crocker showed the greatest inhibition, whereas this sarcoma was very slightly affected by adenylic or guanylic acid.

The possibility that the inhibitory action of 4-aminouracil may be due to the utilization of this compound in some synthetic process in cell metabolism leading to the formation of a purine demands further investigation. In this connection it is significant that 4-methyl uracil and 4-amino-5-methyl uracil, in which purine synthesis is blocked by the methyl group, have been found to have no inhibitory action on sarcoma growth, and the marked difference in the actions of 4-amino-uracil and uracil itself on tumour development is worthy of emphasis. This difference in behaviour of these compounds is further shown by the fact that inhibition of tumour growth was noted when guanylic acid and 4-aminouracil were injected together in grafted mice, whereas it had been previously observed that guanylic and uridylic acids were mutually antagonistic, and that their respective inhibitory and acceleratory actions were cancelled out when injected simultaneously.

Experiments are in progress to examine other aspects of the relationship of purine and pyrimidine compounds to sarcoma growth.

### SUMMARY.

Previous observations regarding the effects of nucleotides and related compounds on the tissues of normal and tumour-bearing mice have been extended. Adenine is found to be highly toxic, but hypoxanthine, whilst inhibiting sarcoma growth, is toxic only on continued dosage. Xanthine has no effect on sarcoma growth, but, like guanylic acid, produces eosinophilia. Of the three pyrimidine bases investigated, orotic acid showed no action on tumour growth; 4:5-diaminouracil was too toxic to allow of continued dosage; 4-aminouracil showed a significant inhibiting effect on grafted tumours, the greatest effect being observed with the Crocker sarcoma.

We are grateful to the British Empire Cancer Campaign for grants defraying the cost of these investigations.

### REFERENCES.

Arvidson, H., Eliasson, N. A., Hammarsten, E., Reichard, P., von Ubisch, H., and Bergstrom, S.—(1949) *J. biol. Chem.*, **179**, 169.

Badger, G. M., Elson, L. A., Haddow, A., Hewett, C. L., and Robinson, A. M.—(1942) *Proc. Roy. Soc.*, B, **130**, 255.

Barakan, T. H., Barker, G. R., Gulland, J. M., and Parsons, L. D.—(1948) *J. Path. Bact.*, **60**, 441.

Brown, G. B., Petermann, Mary L., and Furst, S. S.—(1948) *J. biol. Chem.*, **174**, 1043.

Getler, Helen, Roll, P. M., Tinker, J. F., and Brown, G. B.—(1949) *Ibid.*, **178**, 259.

Parsons, L. D., Gulland, J. M., and Barker, G. R.—(1947) *Symp. Soc. exp. Biol.*, **1**, 179.